# Epidemiology of sleep health and associations with mental health among in-school adolescents in Uganda: A cross-sectional mixed-methods study

**DOI:** 10.1016/j.sleh.2025.12.007

**Published:** 2026-01-20

**Authors:** Prossy Namirembe, Beatrice Nanyonga, Denis Ndekezi, Betty Nyangoma, Rebecca Kyomugisha, Aaron Nyaruhuma, Claudia Ateo, Calvin Robert Rutainama, Katherine A. Thomas, Ratifah Batuusa, Benson Muhindo, Sheila Kasabiiti, Connie Alezuyo, Nambusi Kyegombe, Chris Bonell, Daniel Michelson, Fiona C. Baker, Faith Orchard, Femke Bannink Mbazzi, Helen A. Weiss

**Affiliations:** a MRC/UVRI and LSHTM Uganda Research Unit, Entebbe, Uganda; b Reach A Hand Uganda, Kampala, Uganda; c International Statistics and Epidemiology Group, London School of Hygiene & Tropical Medicine, London, UK; d Ministry of Education and Sports, Kampala, Uganda; e Department of Global Health and Development, London School of Hygiene and Tropical Medicine, London, UK; f Department of Public Health, Environments and Society, London School of Hygiene & Tropical Medicine, London, UK; g Department of Child & Adolescent Psychiatry, Institute of Psychiatry, Psychology & Neuroscience, King’s College London, London, UK; h Center for Health Sciences, SRI International, Menlo Park, California, USA; i School of Psychology, University of East Anglia, Norwich, UK; j Department of Population Health, Faculty of Epidemiology and Population Health, London School of Hygiene & Tropical Medicine, London, UK; k NIHR Maudsley Biomedical Research Centre, South London and Maudsley NHS Foundation Trust and King’s College, London, London, UK; l School of Physiology, University of the Witwatersrand, Johannesburg, South Africa

**Keywords:** Insomnia, Depression, Anxiety, Sleep quality, Students, Africa

## Abstract

**Objectives:**

Few studies have examined sleep health among African adolescents. We aimed to understand sleep health among Ugandan secondary school students.

**Methods:**

We collected quantitative data in two schools through a survey with items on sleep health and insomnia (using the Cleveland Adolescent Sleepiness Questionnaire, Munich Chronotype Questionnaire and Insomnia Severity Index [ISI]) and mental health with the UNICEF Measuring Mental Health Among Adolescents and Young People at the Population Level (MMAPP) tool. We used regression models to assess characteristics associated with ISI score, and of sleep health with depression and anxiety. We conducted focus group discussions and in-depth interviews with students, parents, teachers, and officials. Quantitative and qualitative analyses were guided by the social ecological model of sleep health.

**Results:**

The 358 participants generally reported poor sleep health (assessed by satisfaction, alertness, timing, efficiency and duration), especially among boarding students. The median sleep duration was 5.1 hours (interquartile range 4.2-6.2). Overall, 36 (10.1%) participants screened positive for moderate/severe insomnia (ISI ≥15), with higher prevalence among females than males (12.7% vs. 6.2%; *p* = .05). Qualitative interviews highlighted that individual (knowledge and attitudes), social-cultural (religious beliefs, family dynamics, academic demands, peer pressure), environmental (school and home conditions, technological influences), and societal factors (national school schedule guidelines) influenced sleep patterns. Depression and anxiety were associated with multiple dimensions of poor sleep health.

**Conclusions:**

Ugandan adolescents face substantial sleep challenges, which are associated with poor mental health. Evidence-based interventions should be adapted for specific social-ecological contexts to improve sleep and mental health in this population.

## Introduction

Sleep health is critical for adolescents’ physical, cognitive, and emotional development.^1^ Sleep health is defined as a multi-dimensional pattern of sleep-wakefulness, adapted to individual social and environmental demands, that promotes physical and mental health.^1,2^ In adolescents, sleep health is influenced by multiple socio-ecological factors, across individual, social and structural levels^3,4^ which align with a socio-ecological model (SEM).^5^ Insomnia is a specific sleep disorder defined by recurrent difficulties falling asleep and/or staying asleep, resulting in daytime difficulties such as fatigue, poor concentration and mood disturbance.^6^ Insomnia prevalence is estimated at 4%-39% in adolescents, with differences reflecting the diagnostic criteria or tools used, demographic, geographic and cultural characteristics.^4,7^ Insomnia prevalence tends to increase during adolescence, and is higher amongst females than males after the onset of puberty.^4^

Adolescents who experience poor sleep health often face difficulties with emotional regulation, impaired cognitive function, and heightened stress responses,^4,8^ which may increase the risk of mood disorders.^9^ Disrupted sleep patterns interfere with the ability to process emotions effectively, creating a negative cycle that further disrupts sleep.^9^ Around half of adolescents with insomnia are reported to have co-morbid mental health disorders, with bidirectional associations between insomnia, anxiety, and depression, as well as suicidal thoughts.^4,8^ Biopsychosocial mechanisms, including reduced social interactions and dysfunctional beliefs about sleep, can mediate these relationships.^10,11^

Most data on the causes and consequences of sleep health among adolescents are from high-income settings,^4^ with limited literature from African countries.^12–14^ A narrative review on short sleep duration identified five studies among African adolescents (four from Nigeria and one from South Africa).^12^ The proportion reporting short sleep duration (defined variably as less than 8.25-9.15 hours) ranged from 23%-59% in the African studies.^12^ A review of the relationship between socio-economic position (SEP) and sleep health in African populations identified five studies among adolescents, with low SEP generally associated with shorter sleep duration and greater insomnia symptoms.^14^ In addition to these and other factors affecting sleep in high-income countries (academic demands, extracurricular activities, extensive technology use, peer pressure and family/environmental influences),^15^ adolescents in African countries are more likely to experience early puberty and share sleeping spaces, which may impair sleep quality.^12^ Poor menstrual health (defined as the absence of physical, mental, and social well-being in relation to the menstrual cycle, including access to information, safe materials and facilities, and freedom from stigma and restrictions^16^), is prevalent in many African settings, and can also impact sleep in female adolescents.^17^ Religious practices, such as early morning prayers, communal gatherings, and fasting during school terms can adversely affect sleep, although this has not been studied in Africa.^18^

The aim of this paper is to understand the epidemiology of sleep health among Ugandan secondary school students and to assess the associations of sleep health with depression and anxiety. Specific objectives are to (1) describe multiple dimensions of sleep health among secondary school students in Wakiso District, Uganda based on the SEM of sleep health; (2) estimate the prevalence of insomnia and its relationship with individual, social and societal factors; and (3) estimate the associations of depression and anxiety with sleep health in this population. Approximately 45% of Ugandan secondary schools offer both day and boarding options.^19^ In this paper, we specifically compare day and boarding students, to capture the different social and physical experiences which may influence sleep health.

## Participants and methods

### Study design and population

We report baseline findings from a feasibility and acceptability study delivering a tiered school-based sleep health intervention in two Ugandan secondary schools in Wakiso District, near the capital Kampala. We adopted a sequential mixed-methods design, with an initial cross-sectional quantitative survey informing a subsequent qualitative study to deepen understanding of key issues and patterns that emerged from the survey data. We enrolled study participants from secondary schools with whom we had existing relationships through their prior involvement in a school-based cluster-randomized trial of a menstrual health intervention (MENISCUS trial).^20^ Inclusion criteria for schools were mixed-sex schools in Wakiso District documented as having both day and boarding students, and approximately 40 Secondary 3 (equivalent to the third year of a 4-year secondary Ordinary level education) female students. This size was based on MENISCUS trial data.^20^ Of the four eligible schools in Wakiso district, one school was excluded as it was not within practical reach of the research base in Entebbe. The headteachers in the remaining three schools were contacted and asked to confirm their willingness for their school to participate. Two provided consent and were recruited. These schools were privately owned (rather than Government) and faith-based (one Christian and one Muslim). Parents often select schools based on their religious foundations and perceived moral guidance. The schools primarily catered to students from middle-income households, with tuition fees moderately higher than in nearby government schools, but they are not high-cost institutions and have a similar socio-economic status as 42 other schools included in the MENISCUS trial.^20^ All students in Secondary 2 or 3 in May 2024 were eligible for participation.

### Informed consent

We sought written informed consent from (1) parents/guardians of students aged less than 18 years, followed by electronic informed assent from students whose parents had consented; (2) students aged ≥18 years; and (3) teachers, parents and district officials who participated in the qualitative interviews.

### Ethics approval

Ethics approval was granted by the Uganda Virus Research Institute Research & Ethics Committee (GC/127/819), the Uganda National Council of Science and Technology (HS1525ES), and the London School of Hygiene & Tropical Medicine interventional research ethics committee (22952).

### Data collection methods

We conducted a cross-sectional survey from July 7-August 23, 2024. In addition to socio-demographic and puberty development^21^ items, we collected data on sleep health from the Cleveland Adolescent Sleepiness Questionnaire (CASQ),^22^ Insomnia Severity Index (ISI)^23,24^ and Munich ChronoType Questionnaire (MCTQ)^25^ (Supplementary Table 1). We assessed sleep-related cognitions (Dysfunctional Beliefs and Attitudes about Sleep [DBAS]),^26,27^ and depression and anxiety symptoms using the 25-item UNICEF Measuring Mental Health Among Adolescents and Young People at the Population Level (MMAPP tool Domains 1 and 2; Supplementary Table 2^28^), which provide scores equivalent to the Patient Health Questionnaire-9 [PHQ-9] score for depression and Generalized Anxiety Disorder Assessment-7 [GAD-7] score for anxiety. Depression and anxiety “caseness” were defined as screening positive on items 1-4 (depression) or 5-10 (anxiety), and a cut-off score of ≥18 on the sum of 15 depression- or 13 anxiety-specific MMAPP questions respectively (UNICEF: The MMAPP Toolkit: Operational Guidelines and Protocols (Supplementary Table 2^28^)).

We undertook cognitive testing of survey tools not previously used in this setting (MMAP, DBAS, MCTQ) with 8 students, to ensure cultural appropriateness, clarity, and relevance. This led to minor adaptations to tools, to optimize comprehension and validity. The survey was self-administered on tablets using Open Data Kit (ODK) software, except for the MCTQ, as cognitive testing informed us that administering this tool by trained researchers improved accuracy in these more complex responses on sleep patterns and timing (sleep onset, wake times).

For the qualitative data collection we purposively selected eligible participants in Secondary 2 and 3, 13-19 years regardless of insomnia status. Trained researchers conducted focus group discussions (FGDs) and semi-structured in-depth interviews (IDIs) with students, parents, teachers, and district officials in August and September 2024, in a private location at the school. Topic guides focused on understanding the sleep environment, knowledge and perceptions of sleep. Thematic saturation was achieved after conducting 34 IDIs (2 head-teachers, 2 matrons, 10 female students, 10 male students, 8 parents and 2 District-level officials). We conducted four FGDs with 8 students in each to confirm the themes and insights obtained from the initial IDIs. Individuals who took part in the FGDs were distinct from those in the IDIs, allowing FGDs to validate and refine themes emerging from the interviews. Interviews were conducted in English or Luganda (chosen by the participant). IDIs lasted 35-50 minutes, and FGDs 120-140 minutes.

### Data analysis

**Fig. 1:**
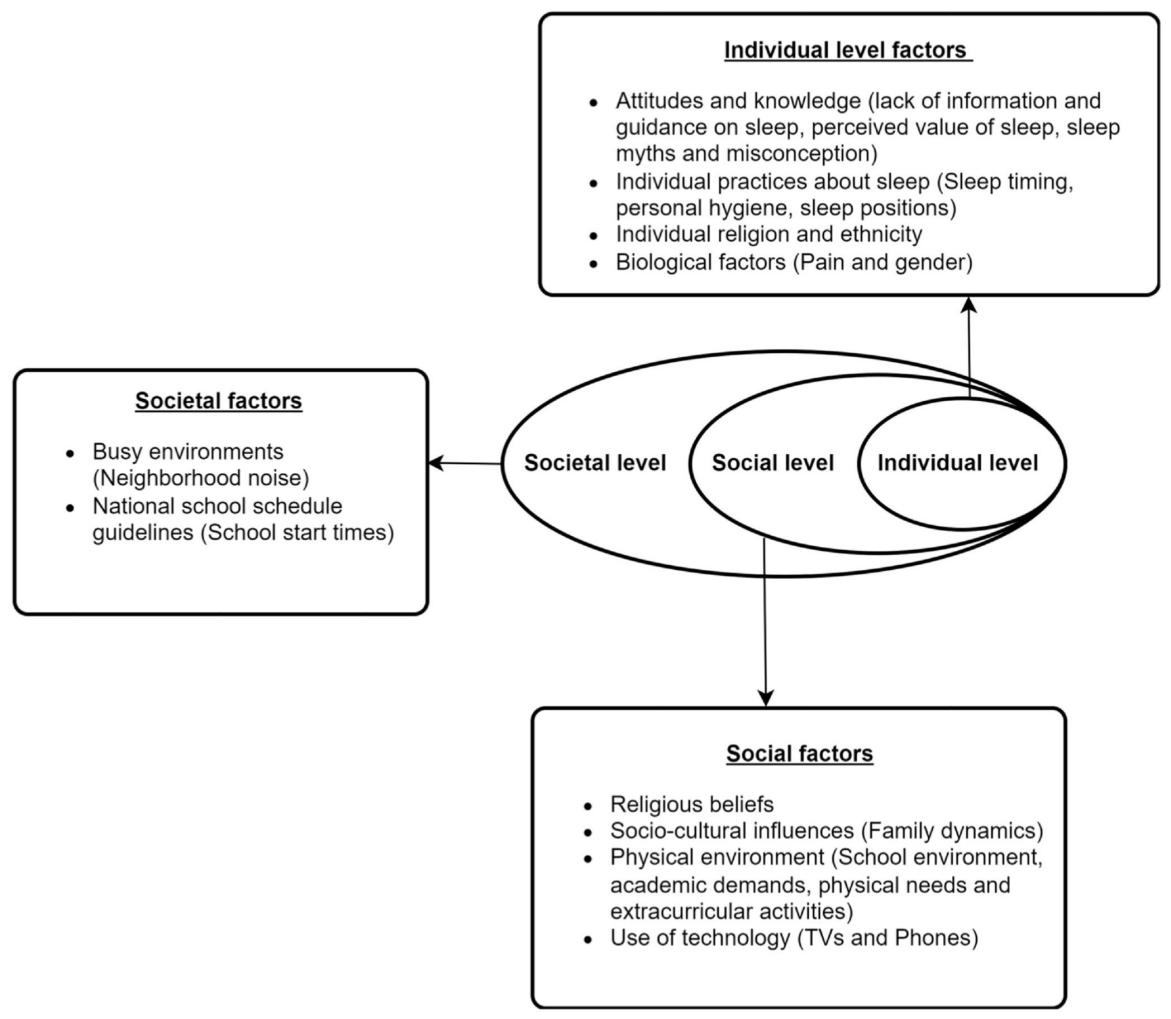
Socio-ecological model for sleep health^5^

**Fig. 2:**
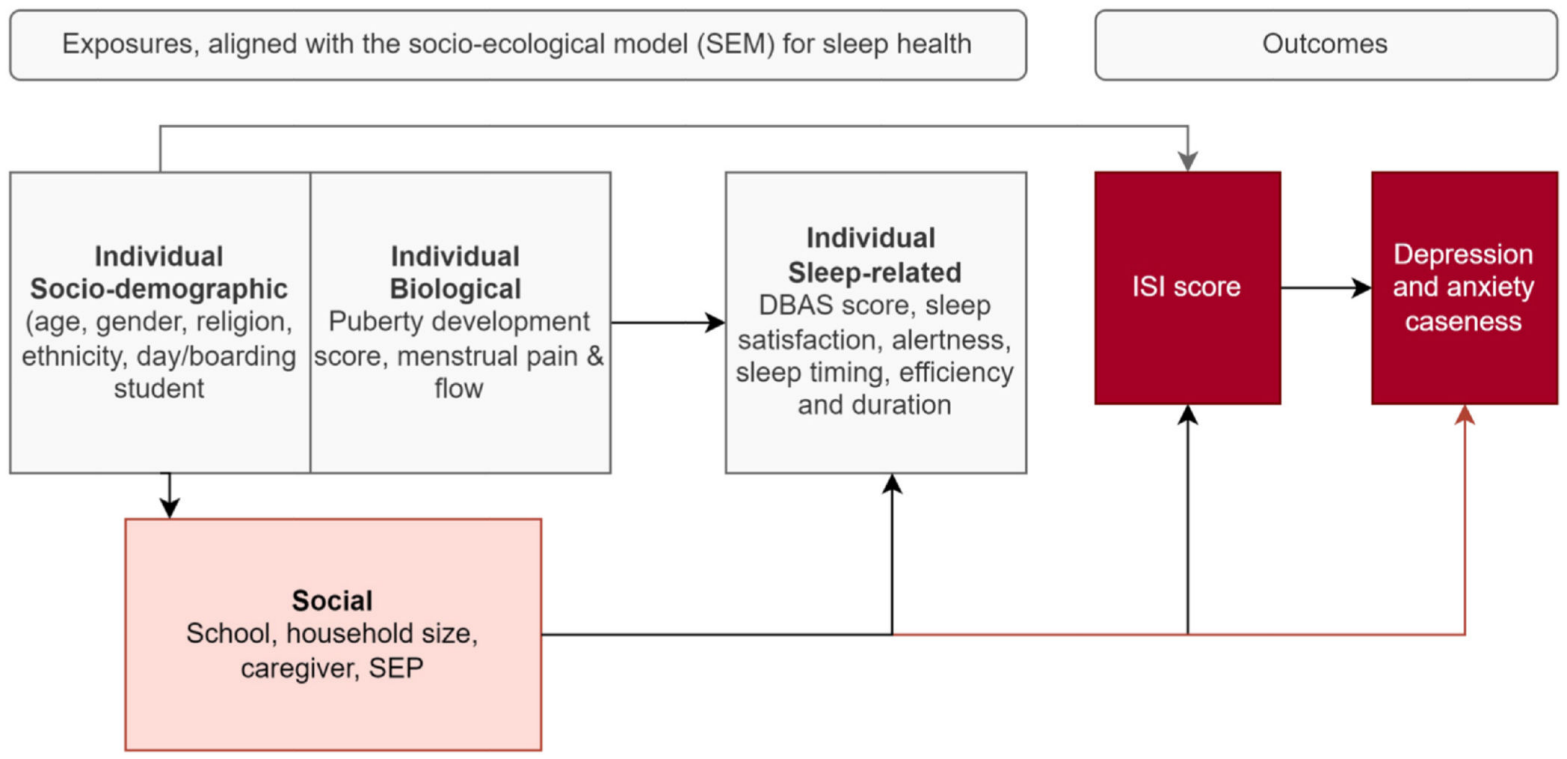
Conceptual framework for quantitative analyses, based on the SEM

We used the Socio-Ecological-Model (SEM) of sleep health^5^ (Fig. 1) to inform a conceptual framework for the analyses of quantitative (Fig. 2), and qualitative data. The SEM conceptualizes how individuals interact with and are influenced by their environment^29^ and has been modified to examine how individual (mental health, and lifestyle habits), social (family dynamics, peer relationships, and school environments), and societal factors (socioeconomic status, cultural norms, and access to healthcare) interact to impact sleep outcomes.^1,3,30^

We conducted descriptive analyses of sleep health stratified by day/boarding status of students, and by sex, as females are known to have poorer sleep health than males, and we hypothesized that boarding students may have poorer sleep than day students. The outcomes for regression analyses were (1) insomnia symptoms (ISI score), (2) depression (MMAPP depression caseness), and (3) anxiety (MMAPP anxiety caseness) using a hierarchical model (Fig. 2), adjusting for school as a fixed effect. We assessed the association of insomnia with individual-level demographic (age, sex, religion, ethnicity, day/boarding student), individual-level biological (puberty development score, menstrual flow and pain (female participants only)) and social-level variables (primary caregiver, school, relative SEP, household size) by adjusting each exposure for each other. Estimates for highly correlated variables (i.e., school and religion; menstrual flow and menstrual pain), were not adjusted for each other. We then assessed the association between sleep-related factors and insomnia adjusted for these individual and social factors. Finally, we assessed associations of mental health outcomes with insomnia category (no insomnia: ISI 0-7; mild insomnia: ISI 8-14; moderate/severe insomnia: ISI ≥15) and other measures of sleep health, adjusted for individual and social factors (Fig. 2). We used linear regression models to estimate adjusted mean differences (aMD) and 95% confidence intervals (CI) for the associations of ISI score. We used logistic regression to estimate adjusted odds ratios (aOR) and 95%CIs associated with depression and anxiety caseness, respectively. Analyses were conducted using Stata version 18.0.

Results from the quantitative data analysis informed the design and focus of the qualitative study. Qualitative interviews were audio-recorded and transcribed verbatim, and interviews conducted in Luganda were translated into English. We used a thematic analysis approach^29^ following the SEM of sleep (Fig. 1). We initiated the coding process with a subset of four transcripts, aiming to uncover and identify prominent and recurring patterns and themes. The initial codes and coding framework were reviewed and refined, resulting in a revised codebook. Axial coding was then used to organize and link related codes into broader themes. We indexed the data and iteratively developed higher-level categories to clarify and refine their meaning in relation to the research questions. The qualitative findings were integrated with the survey results for final interpretation.

## Results

### Sleep health characteristics of participants

Of the 366 students eligible for participation, 358 (97.8%) provided parental consent and/or student assent. The mean age was 15.8 years (standard deviation [SD] = 1.22, range 12-20 years). Most participants were boarding students (64.0%) and were female (59.5%) (Table 1).

Overall, 252 (70.4%) participants reported not being satisfied with their current sleep pattern, and 95 (26.5%) reported being “much” or “very much” worried about their sleep. Most students (286, 79.9%) scored poorly for alertness (CASQ score > 10, indicating an average score of at least sometimes experiencing daytime sleepiness on each of the 5 items), and timing (59.8% had a chronotype (mid-sleep time) of 3 AM or later). The median sleep efficiency (assessed from the MCTQ) was 86.7% (77.4%-92.8%) and was slightly lower on schooldays than on non-schooldays (86.4% vs. 92.1%). The median sleep duration was 4.8 hours on schooldays (IQR 3.9-6.1 hours), and 6.5 hours on non-schooldays (IQR 4.9-8.2), with only 46 (12.8%) students reporting 7-11 hours of sleep on schooldays, and 138 (38.5%) on non-schooldays.

In general, sleep health was poorer among boarding students than day students (Table 2). Sleep satisfaction and alertness were poorer among female than male students, but there was little difference in sleep timing, efficiency or duration by sex (Table 2).

The overall mean ISI score was 8.66 (SD = 4.28), with 150 (41.9%) participating reporting no insomnia symptoms (ISI < 8), 172 (48.0%) mild insomnia (ISI 8-14), 35 (9.8%) moderate insomnia (ISI 15-21) and one severe insomnia symptoms (ISI ≥22). Overall, 45 participants (12.6%) reported always having problems falling asleep, problems sleeping well or problems sleeping too much in the past 2 weeks. In the survey, the most common reason given for a poor night’s sleep was a heavy school timetable (32.9%), followed by noise (19.0%), anxiety (13.3%), temperature (13.0%) and pain (12.1%). The school timetable was more commonly cited among boarding than day students, and pain more often cited by females than males (Table 2).

The ISI score was slightly higher among boarding than day students (9.00 vs. 8.07) and among females than males (9.20 vs. 7.88) (Table 2). Boarding students reported reduced satisfaction, more anxiety about sleep, and a perception of impact on daytime functioning, compared with day students. In contrast, females scored higher than males on all 7 items of the ISI (Table 2). The mean DBAS score was 22.8 (SD 6.33), and the most common dysfunctional belief was that poor sleep will affect the things they need to do the next day (females: 86.4%; males: 73.1%).

### Factors associated with the Insomnia Severity Index

Table 3 shows associations of the ISI score with individual and social-level factors. After adjustment for potential confounders, ISI scores were higher in females than males (aMD = 1.12, 95%CI 0.19, 2.04), and in boarding students than day students (aMD = 0.93, 95%CI 0.01, 1.86). ISI scores were lower among participants with higher puberty development score (aMD = −2.88, 95%CI −5.46, −0.29; *p*-trend = .05). Among females, those with more severe menstrual pain tended to have higher ISI scores than those with no or mild pain (aMD = 1.45, 95%CI −0.08, 2.97; *p*-trend = .06).

There was strong evidence that the ISI score was higher among participants with greater dysfunctional beliefs and attitudes about sleep (aMD = 4.35, 95%CI 1.77, 6.93 for DBAS score of ≥31 vs. < 10). After adjusting for DBAS and individual and social variables, participants with a higher ISI score reported low alertness, poor sleep efficiency, shorter sleep durations, and less time in bed, but there was no evidence of an association with chronotype (Table 3).

The qualitative data also aligned with the SEM (Fig. 1; Table 4). At individual level, participants reported a lack of knowledge regarding the importance of sleep health. Many viewed sleep as a luxury, a waste of time or an outdated concept, prioritizing productivity over rest. Students received misinformation, such as that sleep is for old people and adolescents must sleep for less hours than older adults. However, participants noted that sufficient sleep is vital for rejuvenating their minds and bodies and enhancing learning capacity and academic performance. A recurring theme that female students reported was that menstrual pain disrupted their sleep, often causing difficulty falling asleep, and frequent awakenings during the night. Daytime napping was frequently mentioned as a coping strategy to manage sleep deprivation resulting from late nights and early wake-up times. Several students described unintentionally dozing off during class due to exhaustion and others mentioned deliberately using short breaks or lunch hours to rest.

Personal religious beliefs shaped sleep practices (Table 4). Students reported that their sleep positions were guided by superstitions and religious beliefs, such as girls being prohibited from sleeping on their backs (believed to make them susceptible to male demons). Boys had been warned against sleeping facing down, lest they have sexual intercourse with female demons. Muslim students reported being encouraged to sleep on the right side, as sleeping on the left side may lead to bad dreams or fatigue. In addition, certain students stated being advised to sleep on a cold floor or in a wet place, and with cold clothes on their stomach to cool the body. These practices were believed to help induce more comfortable and restful sleep.

At the social level, religious beliefs informed social norms around sleep. Some participants from the Muslim school viewed sleep as a “mini-death,” which made them feel scared or vulnerable. This fear was intensified when they experienced sleep paralysis, making them feel like a "dead body." However, other participants (both Christian and Muslim) emphasized the importance of balancing work and sleep, citing religious teachings that highlight the need for rest and productivity to maintain well-being and a fulfilling life.

Socio-cultural factors impact sleep patterns, including family dynamics. This was especially evident for female students, who reported that societal expectations and responsibilities at home influenced their sleep. Students also highlighted that academic demands impacted their sleep with teachers typically prioritizing performance over well-being, leading to excessive assignments, punishments, and stress. As a result, students were expected to wake early (often at 3:00 AM) to manage their workload. Peer pressure and social interactions (i.e., late-night gossip and games) also reduced sleep, especially among female students, some of whom went to bed at 1-2 AM. Parents reported that students struggled to juggle personal administration roles and schoolwork. After long study sessions, day students return home to tasks requested by their parents, which reduce their sleep time.

Environmental factors disrupted sleep health, including noise, lights, uncomfortable temperatures in overcrowded dormitories, and sharing beds due to limited space and financial constraints (Table 4). Poor living conditions at home, such as leaking roofs and shaking buildings during rain, were also noted. Peer influence contributed to students’ unlimited access to screens (e.g., television, phones), which impacted their sleep patterns. Participants (both day and boarding) mentioned often watching TV from 9 PM to 5 AM, particularly on Saturdays.

Finally, at societal level, school officials claimed that the national curriculum for lower secondary schools (introduced in 2022) included guidelines designed to ensure students have sufficient time for rest. However, many schools fail to adhere to these guidelines due to tight school schedules. Moreover, students in Muslim schools noted that they are obliged to wake up early (3-4 AM) for prayers. Participants in urban or peri-urban areas expressed that they often face disruptions from external night-time noise pollution, such as loudspeakers announcing concerts or machinery from nearby 24-hour factories, which disrupt their sleep.

### Associations between mental health and sleep-related factors

The prevalence of depression and anxiety was 17.6% and 20.1%, respectively. After adjusting for potential confounders, there was strong evidence that depression and anxiety were associated with poor satisfaction with current sleep patterns, and lower sleep efficiency, more dysfunctional beliefs and attitudes, and more severe insomnia (Table 5).

## Discussion

Poor sleep health and insomnia were prevalent among Ugandan secondary school students, and influenced by individual, social, and societal level factors, in line with the SEM of sleep health.^5^ These findings align with research from high-income countries, which has shown that individual determinants (genetics, beliefs, attitudes, behaviors) and social determinants (socio-economic challenges, school start times, and unsafe neighborhood environment), are linked to shorter sleep and poorer sleep quality among adolescents and young adults.^3,5^ Specific challenges seen in the Ugandan students included the very early wake-up times and religious beliefs.

The prevalence of moderate/severe insomnia in our sample population (10.1%) is similar to that in a study of adolescents aged 13-16 in the United States (9.4% using DSM-IV diagnostic criteria).^31^ In our study, prevalence is slightly higher than in the few other studies which have assessed insomnia among adolescents using the ISI. For example, a study of 720 adolescents in Nepal found a prevalence of 24.2% (95%CI 21.1-27.5%) using a cutoff score ≥11^32^ (compared with 31.8% in our sample population), and a study of 1667 adolescents in Hong Kong found a prevalence of 36.8% (95%CI 34.4-39.1%) using an ISI cutoff score of ≥9^33^ (compared with 48.5% in our population).

Participants in our study reported poor sleep health, including sleep duration substantially shorter than the 8-10 hours recommended for adolescents,^34^ and also shorter than in the few other studies among African adolescents.^14^ A study among children at primary day schools in Kampala, Uganda (mean age 10.3+/−2.6 years), found prevalence of 21.7% for sleep disturbances using the Sleep Disturbance Scale for Children, while 23.8% slept less than 8 hours a night.^35^

In our study, multiple dimensions of sleep health were worse among boarding students than day students, although day students had a later chronotype. These patterns likely reflect different social and physical environmental contexts. In Uganda, boarding students follow highly structured schedules with fixed wake-up and sleep times, often resulting in shorter sleep duration and restricted flexibility. In contrast, day students face variable evening routines, household responsibilities, and commuting demands that can delay bedtime and lead to later mid-sleep time. Given that almost half of Ugandan secondary schools include both day and boarding students,^19^ understanding these contextual differences is essential for designing school-based interventions to promote healthy sleep.

As in other studies among post-pubertal adolescents,^4^ insomnia was more prevalent among females than males. This is likely due to factors including menstruation and post-pubertal hormonal fluctuations,^36,37^ genetics,^38^ and greater susceptibility to anxiety and depression,^39^ which in turn heightens the risk of insomnia. The association between insomnia and menstrual pain is consistent with our previous finding among in-school female Ugandan adolescents.^17^ Similarly, a study in the US among post-menarchal adolescent girls (mean age 13.0 years) showed that sleep disturbance was associated with menstrual pain and premenstrual symptom severity.^40^ Beyond these physiological factors, adolescent girls also face social and cultural pressures such as household duties and academic expectations, which can further reduce restorative sleep.^39^ Our study adds to the literature by confirming sex-specific factors within the lived experiences of adolescents in a low-resource East African setting. Menstrual health interventions may therefore have secondary benefits in improving sleep.^20^

The social environment has an important influence on sleep, including school schedules. Our finding indicate that sleep timing and duration were affected by late-night socializing and engagement in extracurricular activities, which supports findings in high-income settings.^4,15^ Teachers prioritized academic performance over sleep, leading to late bedtime and early wake time. This suggests a lack of awareness of the benefits of sleep on academic performance. In contrast, a systematic review of 11 qualitative studies (in high-income countries)^15^ concluded that school nurses and teachers provided support in addressing sleep issues and promoting healthy sleep habits among students. The contrasting results emphasize the need for context-specific understanding and interventions to address sleep health in students.

The physical environment was also critical in determining sleep health. Students reported overcrowding, bed sharing, uncomfortable temperatures, noise and lights that hindered sleep health, similar to the findings of a study in Ugandan boarding^41^ and primary day schools.^35^ Our results support those from a US-based study which found that modifiable environmental factors such as noise, lighting, and overcrowding can significantly impact adolescent sleep.^42^ In the Ugandan context, the high proportion of boarding students suggests that modifications to the physical sleeping environment may be easier to implement than in other settings.

Depression and anxiety were strongly associated with insomnia and with dysfunctional beliefs and attitudes about sleep. This is consistent with research from high-income settings.^4,8^ Previous research has shown that dysfunctional beliefs reinforce unfavorable expectations and behaviors related to sleep, which in turn lead to sleep disorders.^10,43^ In our study, depression and anxiety were not associated with sleep duration on either schooldays or non-school-days, supporting previous findings that sleep quality is more closely associated with mental health outcomes than sleep quantity.^44^

Strengths of this study include the use of validated tools (pilot-tested among Ugandan students to ensure relevance and comprehension), a mixed-methods approach to assess multiple-dimensions of sleep health and insomnia, and a high response rate that minimized selection bias. Inclusion of both boarding and day students contributes to the sparse literature comparing sleep health in these groups. Our choice of the ISI was guided by its alignment with the current diagnostic criteria of insomnia^6^ and validation in different settings among adolescents.^45,46^ Limitations include a relatively small sample size, which limits the power of the study to detect associations, and the cross-sectional design which precludes causal modeling. Also, by adjusting each exposure for each other we may be adjusting for variables on the causal pathway and under-estimating associations with the outcome. Additionally, the study was limited to two private schools in a peri-urban area, which means the results are not generalizable to all adolescents in Uganda, especially those in rural and government school settings. We did not ask about use of interactive electronic devices such as smartphones, which are increasingly prevalent, as is smartphone addiction among university students in Uganda.^47^ Overuse of these devices may adversely affect sleep, and there is growing evidence that their relationship with poor mental health is mediated by poor sleep among adolescents.^48^ Finally, measurement error of self-reported vs. objective (actigraphy-assessed) sleep duration may depend on ethnicity and socio-economic status, and the extent of this is not known in this population.^49^

## Conclusion

We found a high prevalence of poor sleep health and insomnia in adolescents among school-going students in a Ugandan district, and complex associations between biological, social, and environmental factors influencing sleep health. Depression and anxiety were strongly associated with multiple dimensions of sleep health and with insomnia severity. There is a critical knowledge gap regarding the importance of sleep among key stakeholders, including students, teachers, parents and officials who influence the academic environment. Interventions at each level of the SEM, including CBT-I, revising school timetables, collaborative sleep awareness campaigns, and integration of sleep education sessions into the school curriculum, may create supportive-environments for adolescent sleep-health in schools. Further research through larger, more representative studies and intervention in Uganda and similar settings are needed to evaluate the effectiveness of evidence-based sleep health programs within school and community settings.

## Figures and Tables

**Table 1:** Socio-demographic characteristics by day/boarding status and gender for adolescents at two schools in Uganda

N (%)	Day	Boarding	Female	Male
	129 (36.0%)	229 (64.0%)	213 (59.5%)	145 (40.5%)
*Individual socio-demographic*				
Age in years (mean (SD))	15.79 (1.24)	15.86 (1.22)	15.60 (1.19)	16.18 (1.19)
Age group (y)				
≤15	47 (36.4%)	91 (39.7%)	99 (46.5%)	39 (26.9%)
16	43 (33.3%)	74 (32.3%)	67 (31.5%)	50 (34.5%)
≥17	39 (30.2%)	64 (27.9%)	47 (22.1%)	56 (38.6%)
Sex				
Female	71 (55.0%)	142 (62.0%)	213 (100.0%)	0 (0%)
Male	58 (45.0%)	87 (38.0%)	0 (0%)	145 (100%)
Day/boarding student				
Day	129 (100.0%)	0 (0%)	71 (33.3%)	58 (40.0%)
Boarding	0 (0%)	229 (100%)	142 (66.7%)	87 (60.0%)
Religion				
Christian	55 (42.6%)	77 (33.6%)	84 (39.4%)	48 (33.1%)
Muslim	74 (57.4%)	149 (65.1%)	128 (60.1%)	95 (65.5%)
None/Other	0 (0.0%)	3 (1.3%)	1 (0.5%)	2 (1.4%)
Ethnicity group				
Muganda	101 (78.3%)	168 (73.4%)	151 (70.9%)	118 (81.4%)
Non Muganda	28 (21.7%)	61 (26.6%)	62 (29.1%)	27 (18.6%)
*Social-level variables*				
School				
Islamic school	64 (49.6%)	145 (63.3%)	118 (55.4%)	91 (62.8%)
Christian school	65 (50.4%)	84 (36.7%)	95 (44.6%)	54 (37.2%)
Household size				
> =8	58 (45.0%)	85 (37.1%)	82 (38.5%)	61 (42.1%)
6-7	38 (29.5%)	74 (32.3%)	63 (29.6%)	49 (33.8%)
0-5	33 (25.6%)	70 (30.6%)	68 (31.9%)	35 (24.1%)
Primary caregiver				
Mother	64 (49.6%)	134 (58.5%)	125 (58.7%)	73 (50.3%)
Father	40 (31.0%)	65 (28.4%)	54 (25.4%)	51 (35.2%)
Other	25 (19.4%)	30 (13.1%)	34 (16.0%)	21 (14.5%)
Relative socioeconomic position				
Lowest	27 (20.9%)	46 (20.1%)	44 (20.7%)	29 (20.0%)
Medium-low	26 (20.2%)	45 (19.7%)	45 (21.1%)	26 (17.9%)
Medium	23 (17.8%)	50 (21.8%)	46 (21.6%)	27 (18.6%)
Medium-high	28 (21.7%)	42 (18.3%)	45 (21.1%)	25 (17.2%)
Highest	25 (19.4%)	46 (20.1%)	33 (15.5%)	38 (26.2%)

**Table 2:** Sleep health, insomnia and dysfunction beliefs and attitudes about sleep, by day/boarding status and gender for adolescents at two schools in Uganda

Sleep health characteristics	Day	Boarding	Female	Male
N (%)	129 (36.0%)	229 (64.0%)	213 (59.5%)	145 (40.5%)
*Satisfaction*				
How satisfied are you with your current sleep pattern				
Satisfied (moderately-very)	55 (42.6%)	51 (22.3%)	55 (25.8%)	51 (35.2%)
Dissatisfied	74 (57.4%)	178 (77.7%)	158 (74.2%)	94 (64.8%)
How worried are you about your current sleep?				
Not worried	103 (79.8%)	160 (69.9%)	155 (72.8%)	108 (74.5%)
Worried (somewhat-very)	26 (20.2%)	69 (30.1%)	58 (27.2%)	37 (25.5%)
Alertness				
High (CASQ-school score ≤10)	43 (33.3%)	29 (12.7%)	33 (15.5%)	39 (26.9%)
Low (CASQ-school score > 10)	86 (66.7%)	200 (87.3%)	180 (84.5%)	106 (73.1%)
Mid-sleep time (chronotype)				
Good (< 3 AM)	33 (25.6%)	111 (48.5%)	92 (43.4%)	52 (35.9%)
Poor (≥3 AM)	96 (74.4%)	118 (51.5%)	121 (56.8%)	93 (64.1%)
Sleep efficiency				
Median (IQR)	89% (80-94)	86% (77-91)	86% (77-93)	88% (78-93)
Schooldays				
Good (efficiency ≥85%)	80 (62.0%)	118 (51.5%)	113 (53.1%)	85 (58.6%)
Poor (efficiency < 85%)	49 (38.0%)	111 (48.5%)	100 (46.9%)	60 (41.4%)
Non-schooldays				
Good (efficiency ≥85%)	100 (77.5%)	158 (69.0%)	152 (71.4%)	106 (73.1%)
Poor (efficiency < 85%)	29 (22.5%)	71 (31.0%)	61 (28.6%)	39 (26.9%)
Sleep duration (median (IQR))				
Average weekly sleep duration	6.3 (5.4-7.3)	4.6 (3.9-5.5)	5.1 (4.1-6.2)	5.1 (4.3-6.2)
*Schooldays*				
Sleep duration schooldays	6.3 (5.1-7.2)	4.4 (3.7-5.3)	4.7 (3.8-6.2)	5.0 (4.0-6.0)
Good (7-11 h)	36 (27.9%)	10 (4.4%)	28 (13.1%)	18 (12.4%)
Poor(< 7 or > =12 h)	93 (72.1%)	219 (95.6%)	185 (86.9%)	127 (87.6%)
*Non schooldays*				
Sleep duration non-schooldays	7.9 (6.3-9.2)	5.7 (4.6-7.3)	6.5 (4.9-8.0)	6.3 (4.9-8.5)
Good (7-11 h)	78 (60.5%)	60 (26.2%)	78 (36.6%)	60 (41.4%)
Poor(< 7 or > =12 h)	51 (39.5%)	169 (73.8%)	135 (63.4%)	85 (58.6%)
What is the most common reason you typically have a poor night sleep?				
Heavy school timetable	28 (22.8%)	86 (38.4%)	37 (17.8%)	29 (20.9%)
It is too noisy	21 (17.1%)	45 (20.1%)	10 (4.8%)	12 (8.6%)
It is too hot/cold	25 (20.3%)	20 (8.9%)	26 (12.5%)	19 (13.7%)
I am worried or anxious	22 (17.9%)	24 (10.7%)	35 (16.8%)	11 (7.9%)
I am in pain	15 (12.2%)	27 (12.1%)	30 (14.4%)	12 (8.6%)
It is too light	6 (4.9%)	16 (7.1%)	63 (30.3%)	51 (36.7%)
Other	6 (4.9%)	6 (2.7%)	7 (3.4%)	5 (3.6%)
Insomnia-related characteristics				
Insomnia severity index (ISI) score	8.07 (4.65)	9.00 (4.03)	9.20 (4.28)	7.88 (4.17)
ISI category				
No clinical insomnia (0/7)	65 (50.4%)	85 (37.1%)	75 (35.2%)	75 (51.7%)
Subthreshold insomnia (8/14)	53 (41.1%)	119 (52.0%)	111 (52.1%)	61 (42.1%)
Moderate insomnia (15/21)	10 (7.8%)	25 (10.9%)	27 (12.7%)	8 (5.5%)
Severe insomnia (22/28)	1 (0.8%)	0 (0.0%)	0 (0.0%)	1 (0.7%)
To what extent do you consider your sleep to interfere with your daily functioning				
None/mild	92 (71.3%)	146 (63.8%)	136 (63.8%)	102 (70.3%)
Moderate-severe	37 (28.7%)	83 (36.2%)	77 (36.2%)	43 (29.7%)
Dysfunctional Beliefs and Attitudes (DBAS)				
DBAS total score	22.55 (6.38)	23.00 (6.32)	23.71 (6.07)	21.55 (6.52)

**Table 3:** Factors associated with Insomnia Severity Index (ISI) score

	N	ISI score		Unadjusted mean difference (95%CI)	Adjusted mean difference^a^ (95%CI)
		Mean	SD		
*Individual socio-demographic*					
Sex					
Male	145	7.88	4.17	0	0
Female	213	9.20	4.28	1.32 (0.42, 2.22)	1.12 (0.19, 2.04)
Age group					
≤15	138	8.82	4.61	0	0
16	117	7.94	3.84	-0.88 (−1.93, 0.17)	-0.85 (−1.92, 0.22)
≥17^c^	103	9.27	4.22	0.45 (−0.64, 1.54)	0.62 (−0.54, 1.78)
Religion					
Christian	132	8.90	4.63	0	0
Muslim	223	8.47	4.05	-0.43 (−1.35, 0.49)	-0.01 (−0.94, 0.94)
None/Other	3	12.33	3.51	3.43 (−1.47, 8.34)	3.07 (−1.72, 7.86)
Ethnicity group					
Muganda	269	8.28	4.08	0	0
Non Muganda	89	9.83	4.67	1.56 (0.54, 2.57)	1.10 (0.06, 2.15)
Day or Boarding					
Day	129	8.07	4.65	0	0
Boarding	229	9.00	4.03	0.93 (0.00, 1.85)	0.93 (0.01, 1.86)
*Individual biological*					
Puberty Development Score					
Low	212	8.43	4.29	0	0
Medium	135	9.27	4.23	0.83 (−0.09, 1.75)	0.24 (−0.71, 1.19)
High	11	5.64	2.87	-2.80 (−5.38, −0.22)	-2.88 (−5.46, −0.29)
Flow of last menstrual period (LMP)^b^					
Light	50	9.04	4.12	0	0
Moderate	145	9.24	4.29	0.20 (−1.19, 1.60)	0.27 (−1.12, 1.66)
Heavy	13	10.08	5.27	1.04 (−1.61, 3.68)	1.00 (−1.78, 3.78)
Pain severity^b^					
None/Mild pain	59	8.53	4.06	0	0
Moderate pain	78	8.97	4.00	0.45 (−1.00, 1.90)	0.35 (−1.10,1.81)
Severe pain	71	10.14	4.69	1.62 (0.13, 3.10)	1.45 (−0.08, 2.97)
*Social*					
School					
Islamic school	209	8.61	4.15	0	0
Christian school	149	8.74	4.47	0.13 (−0.77, 1.03)	-0.96 (−2.74, 0.84)
Household size					
> =8	143	9.10	4.68	0	0
6-7	112	8.18	4.34	-0.93 (−1.99, 0.13)	-0.93 (−1.98, 0.12)
0-5	103	8.57	3.55	-0.53 (−1.62, 0.55)	-0.83 (−1.93, 0.27)
Primary caregiver					
Mother	198	8.58	4.27	0	0
Father	105	8.53	4.35	-0.05 (−1.06, 0.97)	0.00 (−1.01, 1.01)
Other	55	9.20	4.22	0.62 (−0.67, 1.90)	0.58 (−0.72, 1.88)
Relative SEP					
Lowest	73	7.45	3.41	0	0
Medium-low	71	8.96	3.97	1.51 (0.11, 2.90)	1.38 (0.01, 2.75)
Medium	73	9.18	3.63	1.73 (0.34, 3.11)	1.28 (−0.09, 2.65)
Medium-high	70	9.23	4.25	1.78 (0.38, 3.18)	1.47 (0.08, 2.86)
Highest	71	8.52	5.66	1.07 (−0.32, 2.46)	0.78 (−0.63, 2.18)
*Sleep health-related*					
DBAS score					
< 10	14	5.36	5.09	0	0
11/20	101	7.38	3.75	2.02 (−0.31, 4.34)	2.59 (0.27, 4.91)
21/30	210	9.33	4.05	3.97 (1.72, 6.22)	4.19 (1.96, 6.43)
31/40	33	9.76	5.34	4.40 (1.80, 7.00)	4.35 (1.77, 6.93)
Alertness					
High (CASQ ≤10)	72	6.92	4.03	0	0
Low (CASQ > 10)	286	9.10	4.24	2.19 (1.10, 3.27)	1.47 (0.38, 2.57)
Mid-sleep time (chronotype)					
Good (< 3 AM)	144	8.43	3.72	0	0
Poor (≥3 AM)	214	8.82	4.62	0.39 (−0.52, 1.29)	0.39 (−0.51, 1.29)
Sleep efficiency					
> 85%	205	7.69	3.81	0	0
< 85%	153	9.96	4.54	2.27 (1.40, 3.14)	1.98 (1.14, 2.82)
*Sleep duration*					
Schooldays					
7-11 h	46	6.17	3.55	0	0
< 7 or > =12 h	312	9.03	4.26	2.86 (1.56, 4.15)	2.38 (1.05, 3.72)
Non schooldays					
7-11 h	138	8.08	4.17	0	0
< 7 or > =12 h	220	9.03	4.32	0.95 (0.04, 1.86)	0.77 (−0.17, 1.70)
*Total time in bed*					
Schooldays	92	7.50	4.39		
7-11 h	266	9.06	4.17	0	0
< 7 or > =12 h				1.56 (0.56, 2.57)	1.24 (0.09, 2.39)
Non schooldays	190	8.15	4.09		
7-11 h	168	9.24	4.42	0	0
< 7 or > =12 h	168	9.34	4.36	1.10 (0.21, 1.98)	0.96 (0.05, 1.86)

a Individual and social variables are adjusted for each other (except for school and religion; and menstrual pain and severity which are not adjusted for each other due to collinearity); Sleep-related factors are adjusted for individual and social variables and DBAS score.

b For menstruating females only (N = 208). The question was “During your last menstrual period, how severe was this pain. Would you say it was mild pain, moderate pain, or severe pain?” Participants who reported no pain at last menstrual period were coded as “none” which was then combined with “mild.” Males and non-menstruating females are coded as not applicable but included in models.

c Not adjusted for religion, due to collinearity

**Table 4:** Qualitative findings on sleep health among Ugandan school students

Theme	Sub-theme	Example quotes
*Individual level*		
Knowledge & attitudes about sleep	Limited knowledge	*“People say sleep is for old people because young people must be active, not asleep” (IDI-Female student-14 years)* *“Teachers always say we are young, and our brains are young too, even though we don’t sleep enough, we can still manage”. (IDI-Male student-16 years)*
	Sleep is important	*“I have decreased the time of reading books just because I should sleep. I cannot read much when I am not resting. When I rest enough, my mind will be refreshed to read.” (Female student IDI-16 years)* *“Sleep is very important because it helps us to refresh in the way we understand things. For example, if you learn from morning to evening, then evening preps without enough rest, you find that you cannot understand the concepts because your brain is tired.” FGD-Female students)*
	Sleep and menstruation	*“We as girls sometimes experience stomachaches during menstruation, which makes us not to sleep. Sometimes you stay all night awake, you even cry” (GDG-Female students)*
Sleep practices	Myths and misconception	*“Someone told me if you are looking for good sleep, you first sleep on the cold floor for the body to get cold then you get to bed and cover yourself... Others say you put wet cold clothes on your stomach so that your body becomes cold to have a comfortable sleep.” (IDI-Male student*-17 years) *“What I have known for a very long time, is a child is not allowed to sleep when they are dressed, and girls are not supposed to sleep in a knicker.” (FGD-Female students)*
	Religious beliefs	*“Now us girls sleeping while facing up in Islam is not allowed… Islam says demons can make you pregnant. For boys or men, you don't have to sleep when facing down. What they told us, as a man you will be sleeping with female demons. So, for a girl to get good sleep, you have to face up not face down, also you should sleep on your right hand.” (Female students-FGD)* *“Islam says when you sleep, sleep on your right-hand side…When you sleep on the left-hand side you can affect your heart and even dream about bad dreams, or a person wakes when they are tired. It brings me comfort when I sleep on the right-hand side.” (FGD-Female students)*
	Coping strategies	*“Sometimes I just doze off in class without meaning to — my eyes can’t stay open because I slept late and woke up before 4 a.m. for morning prep.” (Female students-FGD)* *“After lunch, I run to the dormitory to get a short nap before afternoon lessons. If I don’t rest, I end up sleeping in class.” (Male students-FGD)* *“When the teacher leaves, most of us put our heads on the desk to sleep a bit — we are too tired from reading at night.” (Female students-FGD)*
*Social level*		
Religious beliefs	Sleep is mini-death	*“Sheik [Islamic religious leader] scared us that sleep is a brother to death. He said, it is easy for the person to sleep but it is difficult to wake up. I get scared to sleep because of those words… sometimes when I fall asleep and wake up, I feel like I can’t move my body or even a finger. So, when that happens, I assume myself as a dead body.” (Male students-FGD)*
	Sleep and work	*“The Bible also speaks of a day for rest, a time to relax and recharge so that they can work effectively for the rest of the week without being hindered. If you wake up early, make sure you get enough sleep to stay energized and productive.” (Female Student IDI-17 years)*
Family dynamics	Family demands	*“My mum always tells me to wake up early in the morning since am the only girl at home. She says women are not supposed to oversleep. So, by 10:00 pm, she tells me to go to sleep so that I can wake up very early and do housework.” (Female Student IDI-16 years)*
Academic demands	Heavy school timetable	*“Teachers also take books so seriously without minding us to rest. At times you read and feel exhausted and yet you need to wake up early to catch up with the school demands. For example, we are given so many assignments and when you don’t do all of them, you are being beaten so we feel like dropping out.”(Female student IDI-16 years)*
Social interactions	Socializing at night	*“When it comes to the girl’s side, we always come from evening preps at exactly 10 pm but surprisingly when we reach the dormitory, we start jazzing from 10 pm to 1 am and we remember to sleep when it clocks 2 am yet we wake up at 4:00 am.” (Female students-FGD)*
Physical environment	Environmental factors (noise, temperature, housing structures)	*“You can find that sometimes it is very hot for example, these recent days; and the dormitory is very small do you expect us to sleep well in that hot temperature?” (Male student IDI-17 years)* *“When it rains, and you are sleeping, means it is the end of sleep. Because you cannot sleep in a wet place. The building is shaking, you fear that the wall will collapse on you” (Female student IDI-16 years)*
Technological influence	Unlimited access to screens	*“Saturday is the day for entertainment, we watch the television from 9 pm to 5 am, which is what we call “kukeesa” [You watch until you feel tired].” (Female students-FGD)*
*Societal level*		
National school schedule guidelines	Secondary school curriculum	*“The new lower secondary curriculum provides time for the learners to rest and that is why the old curriculum was revised. But schools have timetables where learners wake up at 3 am, students have pressure.” (Inspector of schools-IDI)* *Most times we feel tired, yet we still have to go for evening preps and do housework. We end up sleeping late, yet again we have to wake up at 3:00 am very early for morning preps” (Male student IDI-17 years)*
	Morning and Night payers	*“We leave preps at 9:30 pm to attend prayers, which cause us to sleep very late. We also have Darasa (prayer) very early at 4 am, and even if we want to go to sleep, we can’t. So, I think we do not get enough sleep.” (Female student IDI-16 years)*
24/7 society	Noise	*“Some schools are located very near to the road or town like this school here; sometimes students can be sleeping, and others are moving around announcing the Concerts with those loudspeakers and they end up waking up students.” S1-IDI-HM* *“The noise is always there because we live near a company that makes sausages, so they have those machines that make noise.” S2-IDI-MP-2*

**Table 5:** Sleep health-related factors associated with depression and anxiety

	Depression				Anxiety		
	N	aOR (95%CI)	*p*-value		N	aOR (95%CI)	*p*-value
Total	63 (17.6%)				72 (20.1%)		
*Satisfaction*							
How satisfied are you with your current sleep pattern							
Satisfied	14 (13.2%)	1	.07		15 (14.2%)	1	.04
Dissatisfied	49 (19.4%)	1.95 (0.95, 4.00)			57 (22.6%)	2.10 (1.05, 4.20)	
How worried are you about your current sleep?							
Not worried	37 (14.1%)	1	.001		43 (16.3%)	1	.001
Worried	26 (27.4%)	3.05 (1.59, 5.85)			29 (30.5%)	2.86 (1.54, 5.32)	
Alertness							
High (CASQ^a^ ≤10)	9 (12.5%)	1	.06		13 (18.1%)	1	.54
Low (CASQ^a^ > 10)	54 (18.9%)	2.26 (0.97, 5.26)			59 (20.6%)	1.26 (0.60, 2.63)	
Mid-sleep time (chronotype)							
Good (< 3 AM)	20 (13.9%)	1	.26		22 (15.3%)	1	.13
Poor (≥3 AM)	43 (20.1%)	1.46 (0.75, 2.81)			50 (23.4%)	1.61 (0.87, 2.98)	
Sleep efficiency							
> 85%	25 (12.2%)	1	.001		30 (14.6%)	1	.002
< 85%	38 (24.8%)	3.00 (1.62, 5.59)			42 (27.5%)	2.52 (1.41, 4.53)	
*Sleep duration*							
Schooldays							
7-11 h	7 (14.6%)	1	.04		8 (17.4%)	1	.20
< 7 or ≥12 h	56 (18.1%)	2.83 (1.03, 7.76)			64 (20.5%)	1.81 (0.73, 4.50)	
Non schooldays							
7-11 h	28 (18.8%)	1	.69		25 (18.1%)	1	.11
< 7 or ≥12 h	35 (16.7%)	1.14 (0.59, 2.21)			47 (21.4%)	1.69 (0.89, 3.21)	
DBAS^b^ score			< .001				
< 10	0 (0%)	-			0	-	.004
11/20	11 (10.9%)	1			11 (10.9%)	1	
21/30	37 (17.6%)	1.70 (0.79, 3.69)			47 (22.4%)	2.36 (1.11, 5.02)	
31/40	15 (45.5%)	8.10 (2.78, 23.6)			14 (42.4%)	6.11 (2.15, 17.4)	
Insomnia			< .001				.001
No insomnia	10 (6.7%)	1			11 (7.3%)	1	
Subthreshold	38 (22.1%)	4.80 (2.09, 11.0)			45 (26.2%)	4.81 (2.20, 10.5)	
Moderate	15 (42.9%)	14.46 (4.9, 42.9)			15 (42.9%)	10.6 (3.75, 29.8)	
Severe	0 (0%)	-			1 (100.0%)		

a Cleveland Adolescent Sleepiness Questionnaire.

b Dysfunctional Beliefs and Attitudes about Sleep.

## Data Availability

The data are available on request from LSHTM Data Compass.

## Box

**What was known before this study**

Little is known on sleep health among African adolescents. Existing research tends to use unidimensional measures (e.g., sleep duration) and overlooks the social and cultural context that influence sleep health. There is also little research on associations of sleep health and mental health in African school settings.

**What this study adds**

We apply a socio-ecological framework to explore how biological, social, and environmental factors influence adolescent sleep health in Uganda. By integrating quantitative and qualitative data, we describe context-specific pathways linking poor sleep with depression and anxiety, highlighting the need for comprehensive school-based interventions.
